# Comment on Bai et al. Effect of Eccentric Training with Different Durations, Intensities, and Contraction Velocities on Upper Limb Muscle Strength: A Meta-Analysis. *Life* 2025, *15*, 456

**DOI:** 10.3390/life15121817

**Published:** 2025-11-27

**Authors:** Anneke Hertig-Godeschalk, Yannick Rothacher, Claudio Perret, Fabian Ammann

**Affiliations:** 1 Neuro-Musculoskeletal Functioning and Mobility, Swiss Paraplegic Research, 6207 Nottwil, Switzerland; 2 Functioning Information Reference Lab, Swiss Paraplegic Research, 6207 Nottwil, Switzerland; 3 Faculty of Health Sciences and Medicine, University of Lucerne, 6002 Lucerne, Switzerland; 4 Institute of Sports Medicine, Swiss Paraplegic Centre, 6207 Nottwil, Switzerland

We read the article “Effect of Eccentric Training with Different Durations, Intensities, and Contraction Velocities on Upper Limb Muscle Strength: A Meta-Analysis” by Bai and colleagues [1] with great interest. We thank Bai and colleagues for publishing this meta-analysis, which highlights the positive effects of eccentric training, particularly in protocols of 4–8 weeks involving high-intensity training programs. We appreciate the effort that went into correcting the analysis and presentation of the data after we raised our initial concerns. However, we still have some serious concerns about this article.

Our first concern is the questionable inclusion of studies without a control group. The eligibility criteria list the availability of a control group as an inclusion criterion (“the control group must either undergo the same form of concentric training or remain inactive during the experimental period”). Nevertheless, there are four studies included that do not fulfill this criterion: Schärer et al. [2], Schärer et al. [3], Vetter et al. [4], and Perret et al. [5]. In the corrected publication [1], the authors added that for Perret et al. [5], data from the 1RM bench press were used for the experimental group, while data from a completely different outcome—handgrip strength—were used for the control group. In our opinion, and following appropriate scientific guidelines [6,7], comparing groups using non-comparable outcome measures is methodologically inappropriate and undermines the validity of the findings. Additionally, as our study (Perret et al. [5]) applied an eccentric arm-crank intervention, it is reasonable to assume that the effects extended to handgrip strength due to the training of the entire upper body and cannot be considered “inactive” or “concentric” training. Additionally, the outcome measures used for the control group were not specified for the other three studies without a control group.

Furthermore, the lack of a valid control group cannot be remedied by the use of standardized mean differences, as stated by the authors. We agree with Bai and colleagues that calculating standardized mean differences is a core method in meta-analyses that allows for the comparison of effects measured on different scales or units. Following this method, a difference in means between two groups measured on the same variable is standardized so that it can be compared with the (standardized) difference in means between two groups that were both measured on another variable. However, this basic idea of standardized mean difference does not apply to the studies lacking a control group, and it does not simply allow replacing a missing control group with another measurement of the treatment group. Nevertheless, Bai and colleagues did just that; they calculated a single standardized mean difference by combining two different variables from the same participants (for Perret et al. [5]: 1RM bench press as “treatment” and handgrip strength as “control”). The authors also did not request the raw data from Perret et al. [5], which is considered good practice when performing this type of analysis [6]. Instead, Bai and colleagues estimated the wanted statistics (mean and standard deviation) from the reported ones (median and interquartile range). Additionally, it seems Bai and colleagues assumed independence between pre- and post-intervention measurements. This assumption clearly does not apply to the studies without a control group, as these data represent repeated measures from the same individuals and are therefore expected to be highly correlated. As a result, the standard deviation of the pre–post difference was overestimated in the authors’ calculation, leading to a distorted estimate of the standardized mean difference further down the analysis pipeline. 

We therefore believe that the results reported by Bai and colleagues [1] should be interpreted with caution. We recommend excluding the four studies lacking a control group from the meta-analysis. This would enable a more accurate and valid evaluation of the effects of eccentric training compared to control conditions across different durations and intensities.
